# Oviduct-Specific Expression of Human Neutrophil Defensin 4 in Lentivirally Generated Transgenic Chickens

**DOI:** 10.1371/journal.pone.0127922

**Published:** 2015-05-28

**Authors:** Tongxin Liu, Hanyu Wu, Dainan Cao, Qingyuan Li, Yaqiong Zhang, Ning Li, Xiaoxiang Hu

**Affiliations:** State Key laboratory for Agrobiotechnology, College of Biological Sciences, China Agricultural University, Beijing, China; Duke University Medical Center, UNITED STATES

## Abstract

The expression of oviduct-specific recombinant proteins in transgenic chickens is a promising technology for the production of therapeutic biologics in eggs. In this study, we constructed a lentiviral vector encoding an expression cassette for human neutrophil defensin 4 (HNP4), a compound that displays high activity against *Escherichia coli*, and produced transgenic chickens that expressed the recombinant HNP4 protein in egg whites. After the antimicrobial activity of the recombinant HNP4 protein was tested at the cellular level, a 2.8-kb ovalbumin promoter was used to drive *HNP4* expression specifically in oviduct tissues. From 669 injected eggs, 218 chickens were successfully hatched. Ten G_0_ roosters, with semens identified as positive for the transgene, were mated with wild-type hens to generate G_1_ chickens. From 1,274 total offspring, fifteen G_1_ transgenic chickens were positive for the transgene, which was confirmed by PCR and Southern blotting. The results of the Southern blotting and genome walking indicated that a single copy of the *HNP4* gene was integrated into chromosomes 1, 2, 3, 4, 6 and 24 of the chickens. As expected, *HNP4* expression was restricted to the oviduct tissues, and the levels of both transcriptional and translational *HNP4* expression varied greatly in transgenic chickens with different transgene insertion sites. The amount of HNP4 protein expressed in the eggs of G_1_ and G_2_ heterozygous transgenic chickens ranged from 1.65 μg/ml to 10.18 μg/ml. These results indicated that the production of transgenic chickens that expressed HNP4 protein in egg whites was successful.

## Introduction

The production of recombinant proteins using transgenic animals is a very powerful and promising technique that can be used to yield diverse pharmaceutical proteins, such as hormones [1], human hemoglobin [2], antibodies [3] and other products. Currently, methods for the production of recombinant proteins within the mammary glands of several species, including transgenic goats [4, 5], sheep [6, 7], cattle [8] and pigs [9], are being developed. Additionally, some recombinant pharmaceutical proteins produced by numerous transgenic animals are being applied to clinical use, such as recombinant human antithrombin III [10]. However, these mammalian transgenic systems have several drawbacks that limit their practical application; for example, the process of extracting recombinant proteins from milk is onerous, and the setting systems for transgenic mammals are time-consuming and expensive. By contrast, because of their shorter generation times, appropriate glycosylation and lower cost, transgenic birds are excellent bioreactors for the production of recombinant proteins [11].

However, the unique features of the reproductive system of birds and the fact that their embryonic development occurs in a shelled egg make it difficult to obtain transgenic birds. In recent decades, approaches to obtaining transgenic birds have been improved. By injecting fertilized embryos with a reticuloendotheliosis virus near the blastoderm region, Salter (1986) successfully obtained transgenic chickens that contained exogenous viral DNA [12]. Bosselman (1989) injected a replication-defective reticuloendotheliosis virus beneath the blastoderm of unincubated chicken embryos and found that the viral vectors were heritable in the progeny [13]. Similarly, Harvey (2002), using a replication-deficient retroviral vector based on the avian leukosis virus (ALV), produced transgenic chickens that expressed biologically active β-lactamase in egg whites [14]. However, to date, the efficiency of germ-line transmission of transgenic systems in birds is still very low and requires optimization.

Lentiviruses, a genus of viruses in the family Retroviridae, are characterized by a long incubation period. Lentiviruses deliver a portion of their viral RNA into the DNA of host cells and have the unique ability to infect nondividing cells [15, 16]. Therefore, lentiviruses are recognized as one of the most efficient gene delivery vectors. McGrew (2004) prepared transgenic chickens using the equine infectious anemia virus (EIAV) and found that the efficiency of germ-line transmission was 4–45% [17]. In addition, tissue-specific and constitutive expression of exogenous genes in transgenic chickens has been achieved using lentiviral vectors [18, 19]. At present, preparation of transgenic chickens using lentiviruses is considered feasible and efficient.

Antimicrobial peptides, a critical part of the innate immune system, are found in a wide range of eukaryotic organisms, from humans to insects. Most of these peptides are less than 10 kDa in size [20, 21]. They display broad-spectrum effects and are able to kill bacteria [22], fungi [23], viruses [24] and tumor cells [25]. Importantly, because of their potential for overcoming bacterial resistance to drugs, antimicrobial peptides are increasingly being recognized as promising therapeutics [26]. In mammals, defensins and cathelicidins are two major classes of antimicrobial peptides [27]. One protein of interest that is an antimicrobial peptide is HNP4, which is encoded by the *DEFA4* gene and possesses six disulfide-linked cysteines. The HNP4 peptide is isolated from the azurophil granules of neutrophils, which contain proteins capable of killing bacteria. The mature HNP4 is a small peptide of 33 residues with a molecular weight of 3,715 Da. Compared with other defensins, HNP4 displays more than 100-fold higher activity against *Escherichia coli* and four-fold higher activity against both *Streptococcus faecalis* and *Candida albicans*, and HNP4 is also able to inhibit the HIV virus [24, 28].

In the present study, we used lentiviral vectors to generate transgenic chickens, with the aim of producing chickens with eggs containing the HNP4 protein. Oviduct-specific expression of HNP4 was driven by the 2.8-kb Ova promoter, and the germ-line transmission of the transgene was confirmed. Thus, we successfully generated transgenic chickens that expressed the HNP4 protein in egg whites.

## Materials and Methods

### Ethics statement

The care and use of all chickens in this study was strictly carried out in accordance with State Key laboratory for Agrobiotechnology regulations, and all the operations were approved by the Animal Welfare Committee of China Agricultural University.

### Vector construction

Because the HNP4 precursor displays no activity against bacteria, in this study, the coding sequences of the mature HNP4 were optimized based on the codon bias in the chicken and synthesized by Sangon Biotech (Shanghai, China). The signal peptide of chicken lysozyme was added to the 5’ end of the optimized HNP4 gene for secretion of protein in the oviductal tubular gland cells. In addition, to facilitate the purification of HNP4 protein from egg whites, a His-tag sequence was also introduced at the 3’ end of the *HNP4* sequence by PCR to form the *HNP4-His* gene.

To test the antimicrobial activity of the HNP4 gene *in vitro*, the sequence of the *HNP4-His* gene with *Hin*d III and *Bam*H I sites was cloned into pBud-EGFP, a plasmid modified from pBudCE4.1 (Invitrogen, USA), to form the pBud-HNP4-His-EGFP plasmid.

**Fig 1:**
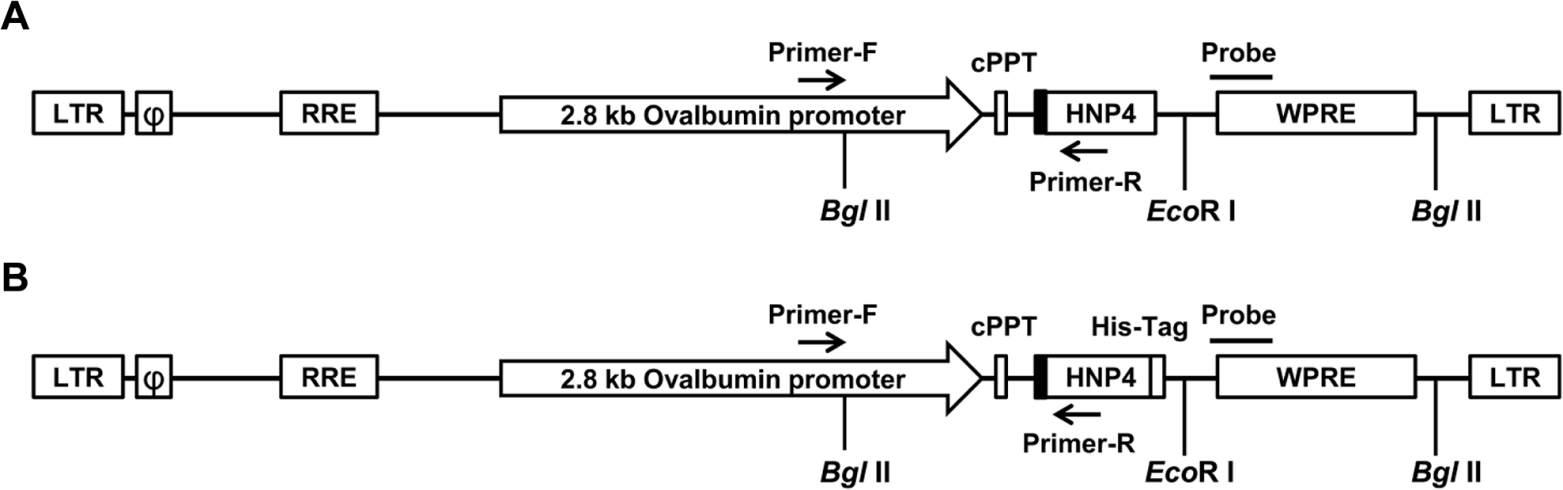
Schematic representation of (A) the pWPXL-Ova-HNP4 and (B) pWPXL-Ova-HNP4-His lentiviral vectors used for oviduct-specific expression in transgenic chickens. LTR, long terminal repeat; ψ, packing signal; RRE, rev response elements; cPPT, central polypurine tract; chicken lysozyme signal peptide (area in black); HNP4, human neutrophil defensin 4; Probe, probe used for Southern blotting; WPRE, woodchuck hepatitis virus posttranscriptional regulatory element. The locations of Primer-F and Primer-R (black arrows) used for screening the transgenic chickens are shown, and the positions of the restriction site *Bgl* II and *Eco*R I used for Southern blot analysis are marked. The figure is not drawn to scale.

The 2.8-kb Ova promoter containing both a steroid-dependent regulatory element (SDRE) and the negative regulatory element (NRE) was amplified from genomic DNA of White Leghorn chickens by PCR and cloned into the pWPXL-GFP vector to replace the EF1-α promoter and to generate the pWPXL-Ova-GFP vector. The HNP4 gene with its *Bam*H I and *Spe* I sites was cloned into the pWPXL-Ova-GFP plasmid by replacing the GFP coding sequence to generate the pWPXL-Ova-HNP4 (Ova-HNP4) lentiviral vector (Fig 1A). The pWPXL-HNP4-His (Ova-HNP4-His) vector was constructed similarly (Fig 1B).

### Cell transfection and antibacterial test

293T cells were transfected with the pBud-HNP4-His-EGFP plasmid using X-tremeGENE 9 DNA transfection reagent (Roche, Switzerland). After incubation of the transfected cells for 24 h, total RNA was extracted from the cells using TRIzol (Invitrogen, USA), and cDNA was prepared using MLV reverse transcription enzymes (Promega, USA). The primers used for RT-PCR amplification are listed in S1 Table. Protein for Western blot analysis was extracted from 293T cells after incubation for 48 h using cell lysis buffer (Beyotime, China).

HNP4-His protein used for antibacterial activity tests was purified on HisPur Ni-NTA resin (Thermo, USA) according to the manufacturer’s instructions. The wafer containing purified HNP4-His protein was placed on an agar plate on which bacteria (DH5α) had been plated. A positive control wafer containing ampicillin and a negative control wafer containing water were also used.

### Lentiviral vector production

The 293FT cells were transfected with three plasmids (Ova-HNP4, psPAX2, pMD2G) to produce lentivirus using the X-tremeGENE 9 DNA transfection reagent (Roche, Switzerland). The viral particles were harvested from the culture medium twice (after 24 h and 48 h of incubation) and then filtered through a 0.22 μm filter. The filtered viral particles were centrifuged at 5000 g/min for 1 h at 4°C in Amicon Ultra-15 centrifugal filters (Millipore, America) and then centrifuged at 50,000 g/min for 2 h at 4°C. The virus particles were then resuspended in virus stored buffer and stored at -80°C. Ova-HNP4-His lentiviral vectors were acquired using the same method. Lentiviral titer was assayed using the Global UItraRapid Lentiviral Titer Kit (System Biosciences, America) following the manufacturer’s instructions.

### Generation and analysis of transgenic chimeric chickens

Transgenic chickens were prepared using surrogate shell methods [29]; all of the White Leghorn donor eggs used in the experiments were from newly fertilized eggs obtained from the North Agricultural Technology company. The subgerminal cavity beneath the blastoderm at stage X was microinjected with 1–2 μl of viral suspension and then placed in the first recipient eggshells and incubated at 37°C at 50%-60% humidity for three days. The embryos were then transferred to a second series of surrogate shells and incubated under the same conditions until hatched.

To identify the transgene in chickens, DNA was extracted from heart, liver, spleen, lung, kidney and muscle of the chicken embryos using a genomic DNA purification kit (Promega, Wizard Genomic DNA Purification) and PCR was performed. To determine the efficiency of lentiviral infection, 15 three-month-old G_0_ hens were randomly slaughtered, and the transgene was identified in the heart, liver, spleen, lung, kidneys and ovaries of these animals.

The amount of DNA used for PCR amplification in G_0_ chimeric chickens was 1 μg. Because the primers used for screening the transgenic chickens did not include the His-tag sequences, the transgenic chickens with the *HNP4* gene or *HNP4-His* gene could be detected by the same primers.

### Generation and analysis of G_1_ and G_2_ hemizygous transgenic chickens

The transgenic chimeric roosters were raised to sexual maturity, and DNA extracted from their semen was screened by PCR. The germ-line positive roosters were crossed with non-transgenic hens to produce G_1_ hemizygous chickens, and the G_1_ chickens were confirmed as hemizygous by PCR. Southern blotting was also performed to confirm the copy number of the gene in the G_1_ animals. Genomic DNA (10 μg) isolated from whole blood of G_1_ transgenic chickens was digested with *Bgl* II and Eco*R* I restriction endonuclease, separated on a 0.7% wt/vol agarose gel, and transferred to a nylon membrane (Roche, Switzerland). The primers used to probe the Southern blots are listed in S1 Table. G_2_ hemizygous chickens were also obtained using the same methods and were confirmed by PCR and Southern blotting.

### Genome Walking of transgenic chickens

The insertion site of the transgene was identified using the Genome Walking Kit (Takara, Japan). The random primers AP1, AP2, AP3, AP4 supplied with the kit and the specific primers SP1, SP2, SP3 designed based on the sequence of the pWPXL-Ova-HNP4-His plasmid were used. The sequences of the designed primers were: SP1, TGTTGGGCACTGACAATTCCG; SP2, CTCAATCCAGCGGACCTTCCTT; and SP3, TCAGACGAGTCGGATCTCCCTT. PCR amplification was performed according to the manufacturer’s (Takara) instructions, and the resulting amplicons were sequenced by BGI (Shenzhen, China). The sequence of the amplicons was searched using BLAST, available through the National Center for Biotechnology Information (NCBI).

### Expression analysis

Total RNA was extracted from the heart, liver, spleen, lung, kidney and oviduct tissue of G_1_ and G_2_ transgenic chickens using TRIzol reagent (Invitrogen, USA). Oviduct tissue of non-transgenic chicken was used as a negative control. Complementary DNA was prepared from 4 μg of RNA using M-MLV reverse transcriptase (Promega, USA). The primers used for RT-PCR amplification are listed in S1 Table.

Goldstar Taq (CWBIO, China) was used for RT-PCR under the following conditions: 95°C for 10 min; 30 cycles of 95°C for 30 s, 55°C for 30 s, and 72°C for 1 min; and 72°C for 5 min. Because the primers used for RT-PCR were not part of the His-tag sequences, the G_1_ and G_2_ transgenic chickens with the *HNP4* and *HNP4-His* genes were analyzed using the same primers.

To validate the observed differences in expression levels, quantitative RT-PCR (qRT-PCR) was performed on tissues from the oviducts of animals of different pedigrees using a LightCycler 480 SYBR Green I Master Kit (Roche, Switzerland) and a LightCycler 480 II real-time PCR detection system (Roche, Switzerland). The hens used for detection were 28 weeks of age, and the PCR conditions were 95°C for 5 min followed by 45 cycles of 95°C for 15 s and 55°C for 1 min. PCR reactions were performed in triplicate for each sample. The △△CT method was used to calculate relative gene expression levels [30]. The internal control used for qRT-PCR was the glyceraldehyde-3-phosphate dehydrogenase (*GAPDH*) gene.

### Immunofluorescence analysis

Oviduct tissue isolated from the slaughtered hens was fixed in 4% paraformaldehyde (PFA) for 24 h, then washed in water for 24 h, followed by paraffin embedding and sectioning at 5 μm on a Thermo HM550 system (Thermo, America). The slides were rehydrated, retrieved for 20 minutes in antigen retrieval solution (Beyotime, China) with microwave heating and blocked in blocking buffer (2% goat serum, 1% BSA, 0.1% Triton-X and 0.05% Tween 20 in PBS) for 2 h. The slides were then incubated in HNP4 antibody solution (1:50 dilution of α-defensin 4 Antibody; Santa Cruz Biotechnology, Inc, USA) overnight at 4°C followed by incubation in secondary antibody solution (1:400 dilution of Alexa Fluor 594 Goat Anti-Rabbit IgG (H+L); Invitrogen, USA) at 37°C for 2 h. The samples were analyzed using a Leica DM5500 B microscope (Leica, Germany).

### Measurement of HNP4 or HNP4-His concentration

To assay the recombinant protein expressed in egg whites, the egg whites were carefully separated from the eggs collected from G_1_ and G_2_ heterozygous chickens in which the transgene was integrated at different sites in the DNA. The amount of HNP4 or HNP4-His in egg whites was assayed using an enzyme-linked immunosorbent assay (ELISA) and a commercially available kit [Defensin Alpha 4, Corticostatin (DEFa4) BioAssay ELISA Kit, USBiological Life Sciences, USA] according to the manufacturer’s instructions.

## Results

### Expression of the *HNP4* gene *in vitro*

**Fig 2:**
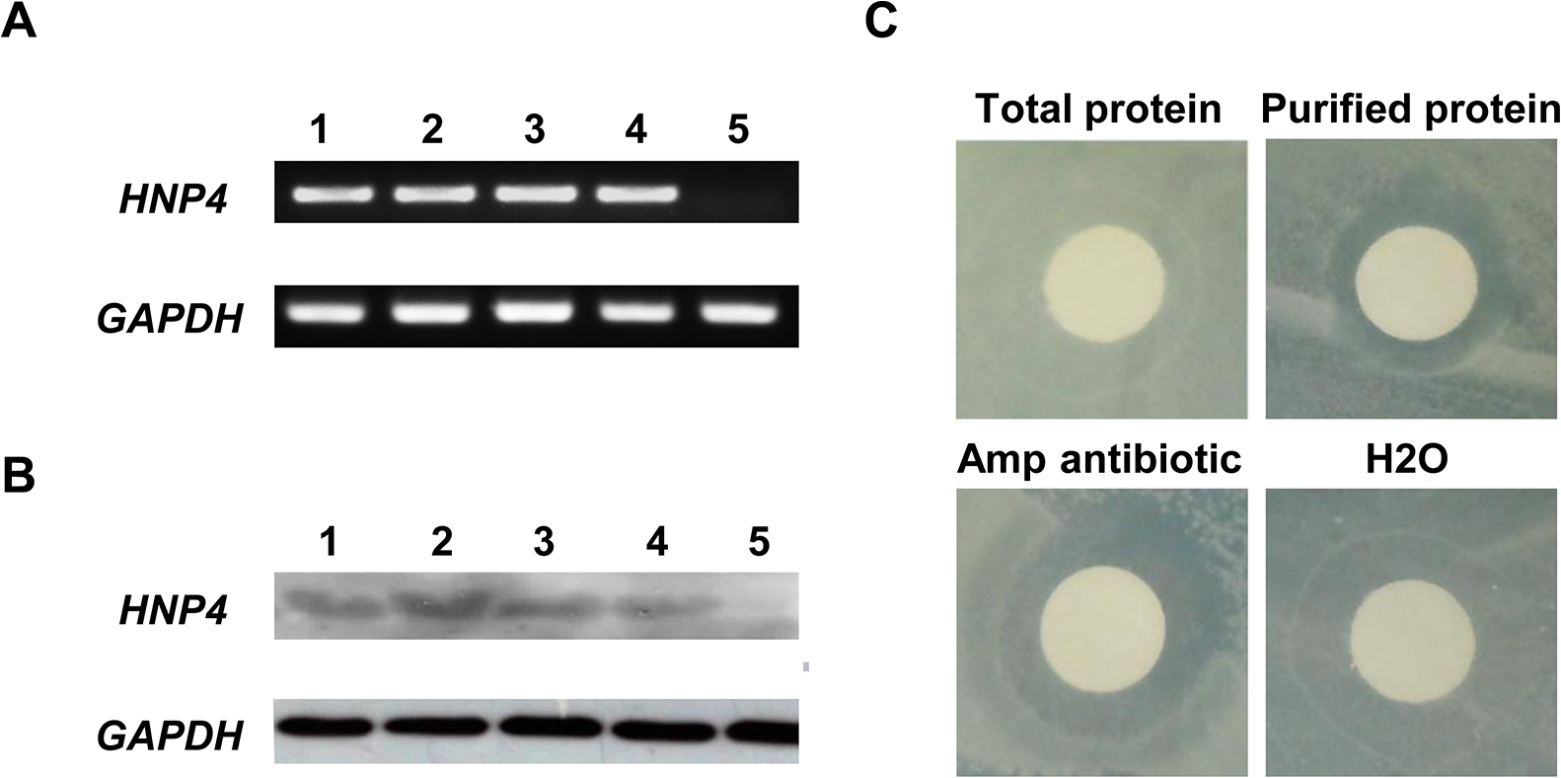
Expression and antimicrobial activity of recombinant HNP4 at the cellular level. A. RT-PCR analysis of the *HNP4* transcripts expressed in transfected 293T cells. Lanes 1–4 represent the 293T cells transfected with the pBud-GFP-HNP4-His vector and lane 5 represents the wild-type 293T cells. B. Western blot analysis of HNP4 protein expression in transfected 293T cells. The results obtained from these samples were consistent with those obtained using samples assayed by RT-PCR. C. Antimicrobial activity of the recombinant HNP4 in vitro. The total protein was extracted from the 293T cells transfected with the pBud-EGFP-HNP4-His plasmid, and the HNP4-His protein was purified from the total protein. Ampicillin was used as a positive control at a concentration of 50 μg/ml, and H_2_O was used as a negative control.

To evaluate the bioactivity of the *HNP4* gene *in vitro* before injection into newly fertilized eggs, the pBud-EGFP-HNP4-His plasmid, modified from the plasmid pBud-EGFP, was transfected into 293T cells. Transfection efficiency was assessed according to the fluorescence of green fluorescent proteins. Both the RT-PCR and Western blot results indicated that the recombinant *HNP4-His* gene was successfully expressed in the transfected 293T cells (Fig 2A and 2B). The activity of the purified HNP4-His protein was assessed using the agar diffusion test. The zone of inhibition indicated that the recombinant HNP4 expressed *in vitro* displayed antibacterial activity (Fig 2C).

### Generation of chimeric chickens expressing the HNP4 gene

The potent promoter of the ovalbumin gene had previously been used to express recombinant proteins in the oviduct tissues of transgenic chickens, but the lengths of the reported Ova promoters varied [19, 31]. In this study, the 2.8-kb Ova promoter containing both a SDRE and a NRE was used to express HNP4 specifically in the oviducts of transgenic chickens [32].

For the generation of transgenic chickens with lentiviral vectors, the lentivirus was pseudotyped with the vesicular stomatitis virus glycoprotein (VSV-G). The woodchuck hepatitis virus posttranscriptional regulatory element (WPRE), which increases both the titer of the virus and the transgenic expression, was also added to our vector [33]. As assayed with the Global UltraRapid Lentiviral Titer Kit, the lentivirus titer was approximately 10^8^–10^11^ IFU/ml. The mature HNP4 protein has only 33 amino acids, and the presence of the His-tag might have affected the antibacterial activity of the HNP4. To avoid this, the lentiviral vectors containing the HNP4 and HNP4-His expression cassettes were injected separately into newly fertilized eggs to produce G_0_ transgenic chickens using surrogate shells.

Because the space available for incubation was limited, we injected the embryos in batches. The total number of eggs injected with the lentivirus was 669; of these, 206 were injected with the vector containing the *HNP4* gene and 463 with the vector containing the *HNP4-His* gene. A total of 218 eggs were hatched into chickens; thus, the average hatching rate was 32.6%. Some of the embryos died during development in the surrogate shells. Six tissues, including heart, liver, spleen, lung, kidney and muscle, were isolated from the dead embryos and then subjected to PCR identification. The results demonstrated that all of the dead embryos were transgenic chimeric chickens (data not shown). To determine the efficiency of lentiviral infection, 15 three-month-old G_0_ hens were slaughtered, and equal amounts of genomic DNA extracted from the heart, liver, spleen, lung, kidney and ovary were analyzed with PCR to assess the level of chimerism of the transgene in the G_0_ hens. The chimerism of the transgene differed among both organs and individuals; all the hens tested were transgenic chimeric animals, as shown in S2 Fig.

### Generation and characterization of G_1_ and G_2_ transgenic chickens

To produce the G_1_ transgenic chickens, the G_0_ chickens were all raised to sexual maturity and the semen samples from the G_0_ roosters were screened by PCR. Four founder roosters (designated G_0_-40, G_0_-48, G_0_-52 and G_0_-54) were transduced with the OVA-HNP4 vector, and six other positive roosters (G_0_-64, G_0_-68, G_0_-70, G_0_-72, G_0_-90 and G_0_-106) were transduced with the OVA-HNP4-His vector; the roosters with semen that tested positive were crossed with non-transgenic hens. Although all 10 of the G_0_ roosters were germ-line-positive, only three (G_0_-40, G_0_-52 and G_0_-72) passed the transgene to the next generation (Table 1).

**Table 1:** Screening of the G_1_ transgenic chickens.

G_0_ roosters carrying the transgene in semen	Number of G_1_ chickens for PCR detection	Number of G_1_ chickens carrying *HNP4* or *HNP4-His*
G_0_-40	167	1
G_0_-48	54	0
G_0_-52	360	5
G_0_-54	243	0
G_0_-64	42	0
G_0_-68	29	0
G_0_-70	22	0
G_0_-72	267	9
G_0_-90	26	0
G_0_-106	64	0

**Fig 3:**
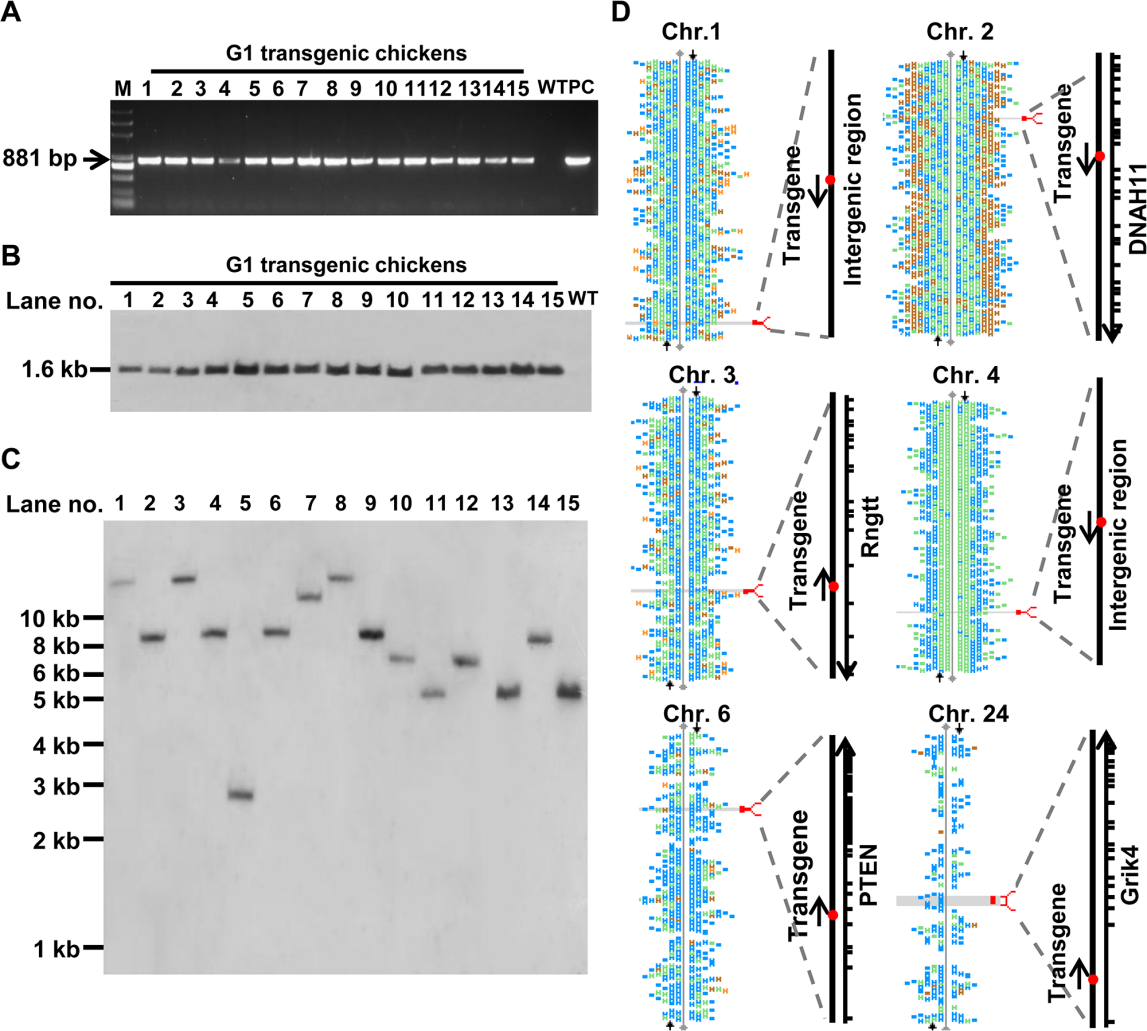
Production and analysis of G_1_ transgenic chickens. A. PCR analysis of the G_1_ transgenic chickens. Lane 1, Marker; Lanes 2–16, DNA samples isolated from the blood of G_1_ transgenic chickens; Lane 17, DNA sample isolated from the blood of nontransgenic chickens; Lane 18, positive control (Ova-HNP4-His plasmid). B. Southern blot analysis of the G_1_ transgenic chickens. Genomic DNA samples extracted from the G_1_ transgenic and wild-type animals were digested with *Bgl* II to confirm the integration of the transgene. Lanes 1–15, DNA samples from the blood of the G_1_ transgenic chickens; Lane 16, DNA sample from the blood of a wild-type chicken. C. Copy number analyzed with Southern blotting. The genomic DNA samples were digested with *Eco*R I to confirm the copy number in the G_1_ transgenic chickens. The results showed six different locations of insertion, and all the G_1_ chickens carried a single copy of the transgene. Lanes 1–15 were the DNA samples extracted from the blood of the G_1_ transgenic chickens. D. Genome walking analysis of the insertion sites. The results indicated that a single copy of the transgene was integrated into chromosomes 1, 2, 3, 4, 6 and 24 of the six chickens.

Genomic DNA isolated from the combs of 167 offspring of rooster G_0_-40 was analyzed with PCR; only one positive chicken was identified. Similarly, five and nine transgenic positive chickens were obtained from the G_0_-52 and G_0_-72 founders, respectively. In total, fifteen G_1_ transgenic chickens—six pullets and nine cockerels—were identified with PCR (Fig 3A); the results were confirmed with Southern blotting. A predicted fragment of 1.6 kb was identified from the G_1_ chickens after digestion with the *Bgl* II restriction endonuclease (Fig 3B), and the junction fragment was also identified after digestion with the *Eco*R I restriction endonuclease (Fig 3C). The Southern blot analysis of the genomic DNA revealed that the G_1_ transgenic chickens carried a single copy of the transgene and that the G_1_ transgenic chickens had six different insertion sites for the transgene.

Genome walking was performed to identify the locations of the transgene in the G_1_ chicken genomes. The transgene of G_1_-6, G_1_-9 and G_1_-28 was integrated at the intergenic region on chromosome 1, whereas in chickens G_1_-7, G_1_-12, G_1_-26, G_1_-31 and G_1_-L19, the transgene was integrated into the thirteenth intron of the dynein axonemal heavy chain 11 (*DNAH11*) gene on chromosome 2. The transgene of G_1_-27 was integrated at the fourteenth intron of the RNA guanylyltransferase (*Rngtt*) gene located on chromosome 3; the transgene of G_1_-46 and G_1_-L5 was integrated at the intergenic location on chromosome 4; the transgene of G_1_-L2, G_1_-L6 and G_1_-L34 was integrated at the fifth intron of the phosphatase and tensin homolog (*PTEN*) gene located on chromosome 6; and the transgene of G_1_-23 was integrated at the first intron of the glutamate receptor ionotropic kainate 4 (*Grik4*) gene located on chromosome 24 (Fig 3D). The results confirmed that the G_1_ chickens had six different insertion sites for the transgene (Table 2). The results of the Southern blot analysis were consistent with this conclusion.

**Table 2:** Genome walking analysis of the G_1_ chickens.

Number	Sex	Vector	Location	Gene
G_1_-6	male	Ova-HNP4-His	chr1:183,382,803	Intergenic
G_1_-9	female	Ova-HNP4-His	chr1:183,382,803	Intergenic
G_1_-28	male	Ova-HNP4-His	chr1:183,382,803	Intergenic
G_1_-7	female	Ova-HNP4-His	chr2:30,615,368	Thirteenth intron in the *DNAH11*
G_1_-12	male	Ova-HNP4-His	chr2:30,615,368	Thirteenth intron in the *DNAH11*
G_1_-26	female	Ova-HNP4-His	chr2:30,615,368	Thirteenth intron in the *DNAH11*
G_1_-31	male	Ova-HNP4-His	chr2:30,615,368	Thirteenth intron in the *DNAH11*
G_1_-L19	male	Ova-HNP4-His	chr2:30,615,368	Thirteenth intron in the *DNAH11*
G_1_-27	male	Ova-HNP4-His	chr3:75,377,333	Fourteenth intron in the *Rngtt*
G_1_-46	female	Ova-HNP4	chr4:70,169,573	Intergenic
G_1_-L5	male	Ova-HNP4	chr4:70,169,573	Intergenic
G_1_-L2	male	Ova-HNP4	chr6:9,103,891	Fifth intron in the *PTEN*
G_1_-L6	female	Ova-HNP4	chr6:9,103,891	Fifth intron in the *PTEN*
G_1_-L34	female	Ova-HNP4	chr6:9,103,891	Fifth intron in the *PTEN*
G_1_-23	male	Ova-HNP4	chr24:3,667,967	First intron in the *Grik4*

Only 10 G_1_ chickens (eight cockerels and two pullets) survived to sexual maturity. The mature G_1_ transgenic chickens were mated with nontransgenic chickens to generate the G_2_ transgenic chickens. The G_2_ chickens were also identified with PCR and Southern blotting, as shown in S4 Fig. In the G_2_ chickens, the positive rate of the population was 0.5, according to Mendelian inheritance. The statistical test for the positive rate was performed with the IBM SPSS statistical software package, followed by t-tests (P > 0.05). There was no significant difference between the positive rate and the population positive rate, which corresponds to the Mendelian inheritance (Table 3).

**Table 3:** Number of G_2_ chickens derived from the G_1_ chickens.

Founder rooster	Total number of offspring	Number of positive offspring	Positive rate
G_1_-12	119	63	0.529
G_1_-L2	72	35	0.486
G_1_-28	145	70	0.482
G_1_-31	136	72	0.529
G_1_-27	70	33	0.471
G_1_-L5	76	36	0.473

### Oviduct-specific expression of HNP4 and HNP4-His in transgenic chickens

**Fig 4:**
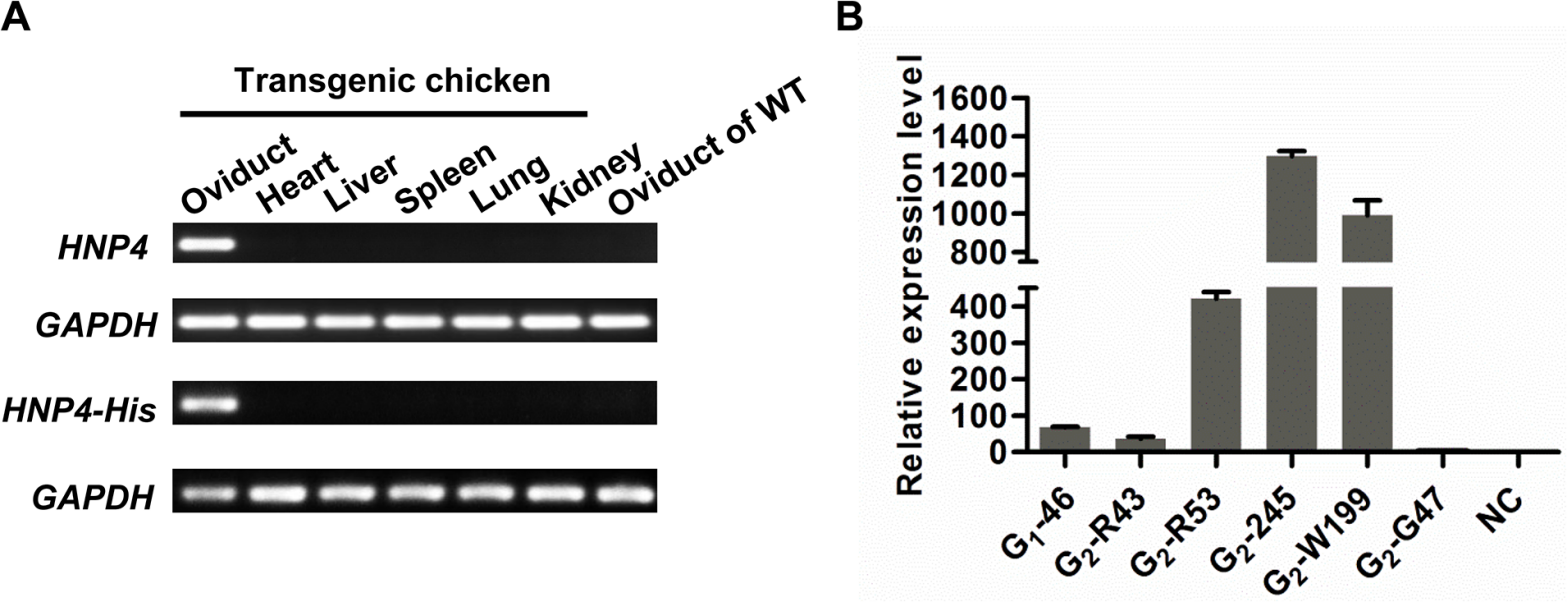
Expression of HNP4 and HNP4-His genes in transgenic chickens. A. RT-PCR analysis of the expression of the HNP4 and HNP4-His genes in the transgenic hens (G_1_-46 with HNP4 and G_1_-7 with HNP4-His). Lanes 1–6 represent the oviduct, heart, liver, spleen, lung, and kidney tissues from the transgenic hen (G_1_), respectively. Lane 7 represents the oviduct tissue from the nontransgenic hens. B. qRT-PCR was used to quantify the transgene in the G_1_ and G_2_ hens with different insertion sites. Column 1 represents the oviduct of G_1_ hen G_1_-46; columns 2–6 represent the oviducts of G_2_ hens G_2_-R43, G_2_-R53, G_2_-245, G_2_-W199, and G_2_-G47, respectively; and NC represents the oviduct of a nontransgenic hen.

Because we used the 2.8-kb fragment of the Ova promoter for production of the HNP4 protein in the egg whites, we expected that HNP4 or HNP4-His would be specifically expressed in the oviduct tissues. The transgenic hens G_1_-46 and G_1_-7 were selected for investigating the expression patterns of *HNP4* or *HNP4-His* transgenes, respectively, at the transcriptional level in six tissues (oviduct, heart, liver, spleen, lung and kidney). As expected, the RT-PCR results indicated that the transgenes were specifically expressed in the oviduct tissue, and no signal was detected in the other tissues or in the wild-type (WT) chickens (Fig 4A).

The RT-PCR results obtained with the G_2_ transgenic chickens G_2_-R201, G_2_-106, and G_2_-246, which were derived from G_1_-46 and G_1_-7, were identical to those obtained with the G_1_ generation chickens. This finding demonstrated that the transgene was specifically expressed in oviduct tissue and was stable in germ-line transmission.

To determine whether the insertion site of the transgene affected the expression of the gene in the oviduct tissues, the qRT-PCR was performed in the oviduct tissues of hens with different insertions for the transgene. We found that the level of *HNP4* expression was highly variable in transgenic chickens with different insertions for the transgene (Fig 4B), demonstrating that the insertion sites significantly influenced the gene expression.

### Recombinant proteins are expressed in the oviduct

**Fig 5:**
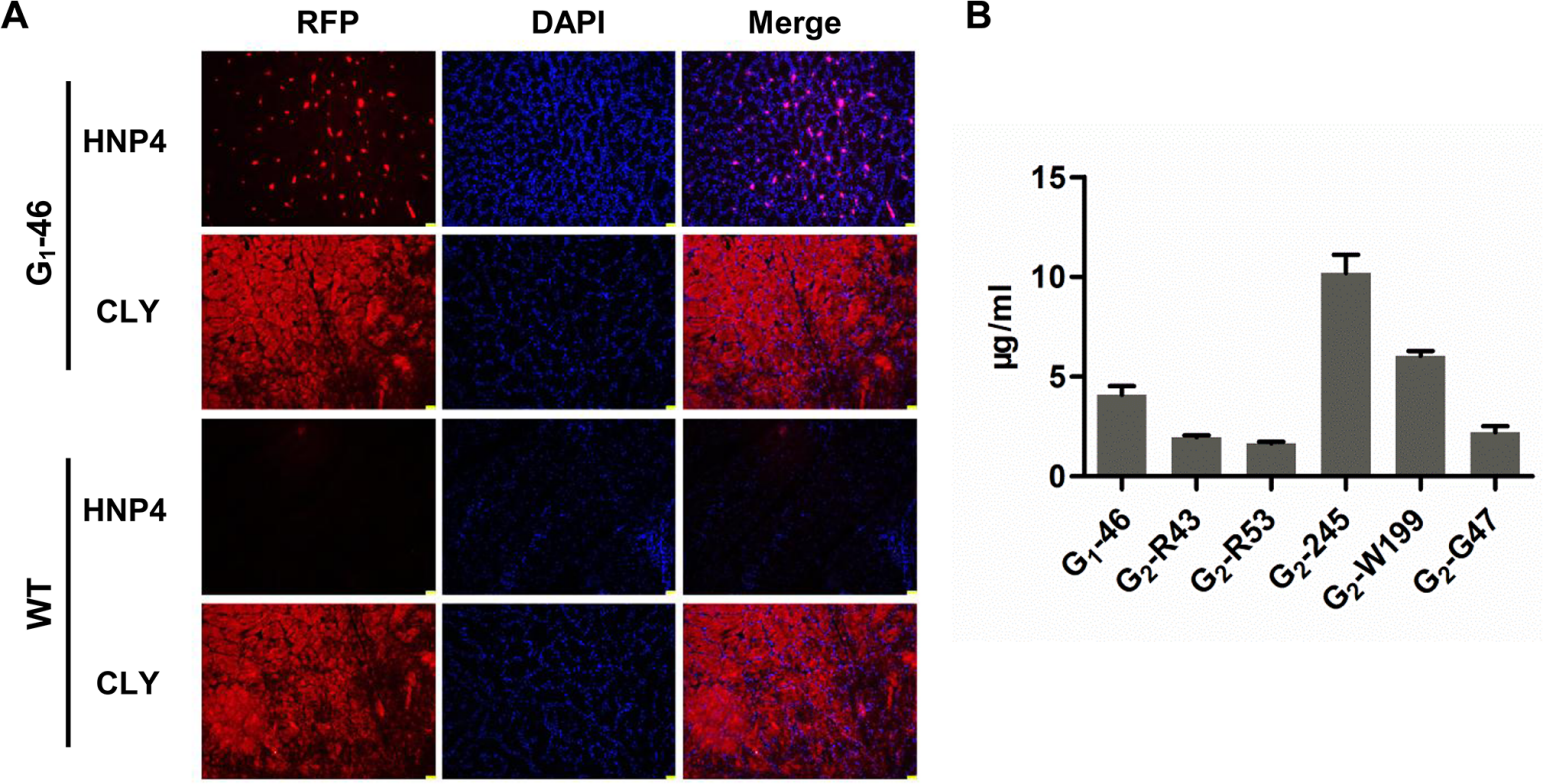
Analysis of the expression of the HNP4 and HNP4-His genes at the translational level. A. Immunofluorescent detection of HNP4 in oviduct sections. HNP4 protein (red) in the oviduct of hen G_1_-46 was visualized in a section of the magnum portion of the oviduct by staining with the HNP4 antibody; the oviduct sections were also stained with the chicken lysozyme antibody (CLY) as a positive control. The oviduct sections of the wild-type (WT) chickens were stained with the HNP4 antibody, but no signal was observed. B. Yields of HNP4 in the egg whites of the G_1_ and G_2_ hemizygous chickens. The egg white solution was assayed by ELISA. The quantities of HNP4 varied greatly in the hens with different insertions, ranging from 1.65 μg/ml to 10.18 μg/ml.

We added chicken lysozyme signal peptides to the 5’ ends of the *HNP4* and *HNP4-His* genes because we expected that the recombinant proteins would be secreted in the egg whites. To localize the recombinant HNP4, immunofluorescence analysis was performed on magnum sections of the oviducts from the G_1_ heterozygous hens. The results obtained for chicken G_1_-46 and wild-type chickens (WT) showed that the HNP4 protein was expressed in the tubular glands of the magnum portion of the oviduct (Fig 5A). The G_2_ heterozygous chickens were also tested, and the results were consistent with those of the G_1_ chickens. These results indicated that the recombinant proteins were expressed in the oviduct.

The yield of the HNP4 or HNP4-His protein in the egg whites of G_1_ and G_2_ heterozygous chickens was measured using the enzyme-linked immunosorbent assay (ELISA), and the eggs were from hens with different insertion sites. All the transgenic chickens produced HNP4 or HNP4-His proteins in the egg whites, and their expression levels in the transgenic chickens with different insertion sites ranged from 1.65 μg/ml to 10.18 μg/ml (Fig 5B). The results showed that recombinant HNP4 was successfully expressed in the egg whites.

## Discussion

Because abuse of antibiotics negatively affects human health, there is an urgent need for new drugs to replace currently used antibiotics. Antimicrobial peptides are considered the best choice for this purpose [26]; however, their application is limited because of the low yield of these peptides using current production methods. One important antimicrobial peptide is HNP4, which is expressed in the granules of neutrophils. Neutrophils defend the host against bacteria and viruses, and HNP4 displays particularly high activity against bacteria [28]. In this study, in an attempt to develop a new method to produce antimicrobial peptides, we produced recombinant HNP4 in egg whites.

In recent years, lentiviral vectors have been thought to be the most effective tool for the production of transgenic birds [17, 19, 34]. It has been shown that genes transduced by lentiviral vectors can be stably transferred through the germ-line. In the present work, we injected a lentiviral vector into 669 embryos, from which 218 chickens were hatched. The average hatch rate was 32.6%, which was lower than the hatch rate cited in previous reports using the surrogate eggshells [35]. Therefore, our approach, which used surrogate eggshells, can be further refined. The positive rate that we detected in G_0_ chickens was as high as 100%, and our results showed that the lentivirus could efficiently infect various tissues, consistent with the characteristics of lentiviruses [15]. Although we obtained a high number of G_0_ chickens, the number of germ-line-positive roosters was very low. One reason for the low positives was that some of the G_0_ roosters died before sexual maturity, and we could not confirm whether the dead roosters were germ-line-positive. Another reason was that the germ-line chimerism was low in the G_0_ chickens. Finally, ten roosters were identified as positive with PCR. Southern blot was also performed on the sperm DNA of G_0_ roosters, but we did not obtain any positive results because of the low chimerism in our sample (data not shown). The efficiency of germ-line transmission was lower (between 0.6% and 3.4%) than that reported previously [17].

Of the ten germ-line-positive roosters that we obtained, only three passed the transgene to the next generation (Table 1). Some of the germ-line-positive roosters (G_0_-48, G_0_-64, G_0_-68, G_0_-70, and G_0_-90) produced very little sperm, possibly because integration of the transgene disrupted the genes associated with testis development. We also found that one chimeric cock produced offspring with different transgene insertion sites, indicating that the lentiviruses had inserted into multiple primordial germ cells.

To obtain oviduct-specific expression of the recombinant HNP4 protein in transgenic chickens, we used the 2.8-kb chicken Ova promoter to drive the expression of the transgene specifically in the tubular gland cells of the oviduct [19]. The promoter contains two regulatory elements, SDRE and NRE. The SDRE, spanning from -892 to -780, increases ovalbumin gene transcription in the presence of four steroid hormones, and the NRE, spanning from -308 to -88, appears to have the dual role of repressing transcription in the absence of steroids and cooperating with the SDRE to activate transcription in the presence of steroids [36]. Although there are several SNPs in the promoter sequence of the chicken ovalbumin gene, no SNPs were present in the core sequence of the Ova promoter (data not shown). As shown by the RT-PCR analysis, expression of the transgene was restricted to the oviducts and was not detected in other tissues, indicating that the 2.8-kb promoter successfully drove transgene expression in the oviduct tissues. In a previous report [19], the 2.8-kb promoter was sufficient for high-level production of recombinant proteins in the chicken oviduct; however, in our study, the quantities of HNP4 and HNP4-His proteins expressed in the egg whites were not as high as those reported previously. The lower levels might be explained by differences in the location of the inserted transgene; it is well known that the location of genes in chromosomes plays a major role in their expression. In our study, we obtained 15 G_1_ heterozygous chickens with six different insertion sites, and the expression levels of *HNP4* varied in chickens with the different insertions, as assayed with qRT-PCR and ELISA (Figs 4B and 5B), indicating that insertion position was the primary factor affecting gene expression in our system. In recent years, preparation of transgenic chickens using primordial germ cells (PGCs) has been considered an efficient method [37], and gene-targeted transgenic chickens have been successfully produced using this method in other laboratories [38, 39]. Gene targeting is the fundamental method to overcome positional effects, and the problem of positional effects in transgenic chickens might be solved in the future with the development of PGC technology.

Gene silencing is one of the major obstacles to the generation of transgenic chickens. In this study, we found no evidence of HNP4 silencing in any generation of the transgenic chickens analyzed. However, we plan to continue to assess transgene expression in successive generations to confirm its stability.

In summary, we have developed a new system for producing recombinant HNP4 protein in egg whites. The biological activity and security of the recombinant HNP4 proteins expressed in the oviducts will be evaluated in future experiments and our study might provide a new clue for producing antimicrobial peptides with oviduct bioreactor.

## Supporting Information

S1 Fig Transfection efficiency of 293T cells. 293T cells were transfected with pBud-EGFP-HNP4-His, and control cells were transfected with an empty vector. GFP was expressed in the transfected cells after incubation for 24 h.
(TIF)

S2 Fig PCR analysis of the partial G_0_ hens. The primers used to screen the transgenic chickens generated an 881-bp PCR product. The figure shows some of the G_0_ hens detected using this method; the G_0_ hens shown in the figure are G_0_-7, G_0_-37, G_0_-38, G_0_-50, G_0_-61, G_0_-62, and G_0_-69. The amount of DNA from G_0_ chickens used for PCR was 1 μg. M, 100-bp ladder; 1, heart; 2, liver; 3, spleen; 4, lung; 5, kidney; and 6, ovary; NC, negative control (oviduct of the wild-type hen); and PC, positive control (plasmid).
(TIF)

S3 Fig Family trees of the transgenic chickens. A. Three G_0_ chimeric chickens (G_0_-72, G_0_-52, and G_0_-40) were crossed with nontransgenic hens to produce the G_1_ hemizygous chickens. In total, 15 G_1_ chickens (G_1_-26, G_1_-7, G_1_-9, G_1_-12, G_1_-28, G_1_-31, G_1_-L19, G_1_-6, G_1_-27, G_1_- 46, G_1_-L6, G_1_-L34, G_1_-L2, G_1_-L5 and G_1_-23) were obtained. B. The G_2_ chickens were obtained using the same methods.
(TIF)

S4 Fig Analysis of the G_2_ transgenic chickens. A. PCR analysis of the G_2_ transgenic chickens. Lane 1, Marker, and Lanes 2–19, DNA samples isolated from the combs of G_2_ transgenic chickens derived from G_1_-positive chickens. B and C. Southern blot analysis of the G_2_ transgenic chickens. “+” represents positive G_2_ chickens, and “-” represents nontransgenic chickens. The genomic DNA samples were digested with *Bgl* II (B) and *Eco*R I (C) to confirm the integration and the copy number of the transgene in the G_1_ transgenic chickens.
(TIF)

S5 Fig Expression of *HNP4* and *HNP4-His* genes in the G_2_ transgenic chickens. RT-PCR analysis of the expression of the HNP4 and HNP4-His genes in the G_2_ transgenic hens (G_2_-R201 with HNP4 and G_2_-106 and G_2_-246 with HNP4-His). Lanes 1–6 represent the tissues of the oviduct, heart, liver, spleen, lung and kidney in the transgenic hens (G_2_), respectively. Lane 7 represents the oviduct tissue in the wild-type hens.
(TIF)

S6 Fig Immunofluorescent detection of HNP4 in the oviduct sections of the G_1_ and G_2_ chickens. HNP4 protein (red) in the oviduct tissues of hens G_2_-R53, G_2_-245, G_1_-7 and G_2_-G47 was visualized in the sections of the magnum portion of the oviducts by staining with the HNP4 antibody.
(TIF)

S1 Table Primers list. (DOCX)

S2 Table Lentiviral vector injection into chicken embryos. (DOCX)
