# Supplementary figures and images for: Oviduct-Specific Expression of Human Neutrophil Defensin 4 in Lentivirally Generated Transgenic Chickens
Source: PLoS One. 2015 May 28;10(5):e0127922. doi: 10.1371/journal.pone.0127922 (PMC4447378; doi:10.1371/journal.pone.0127922)

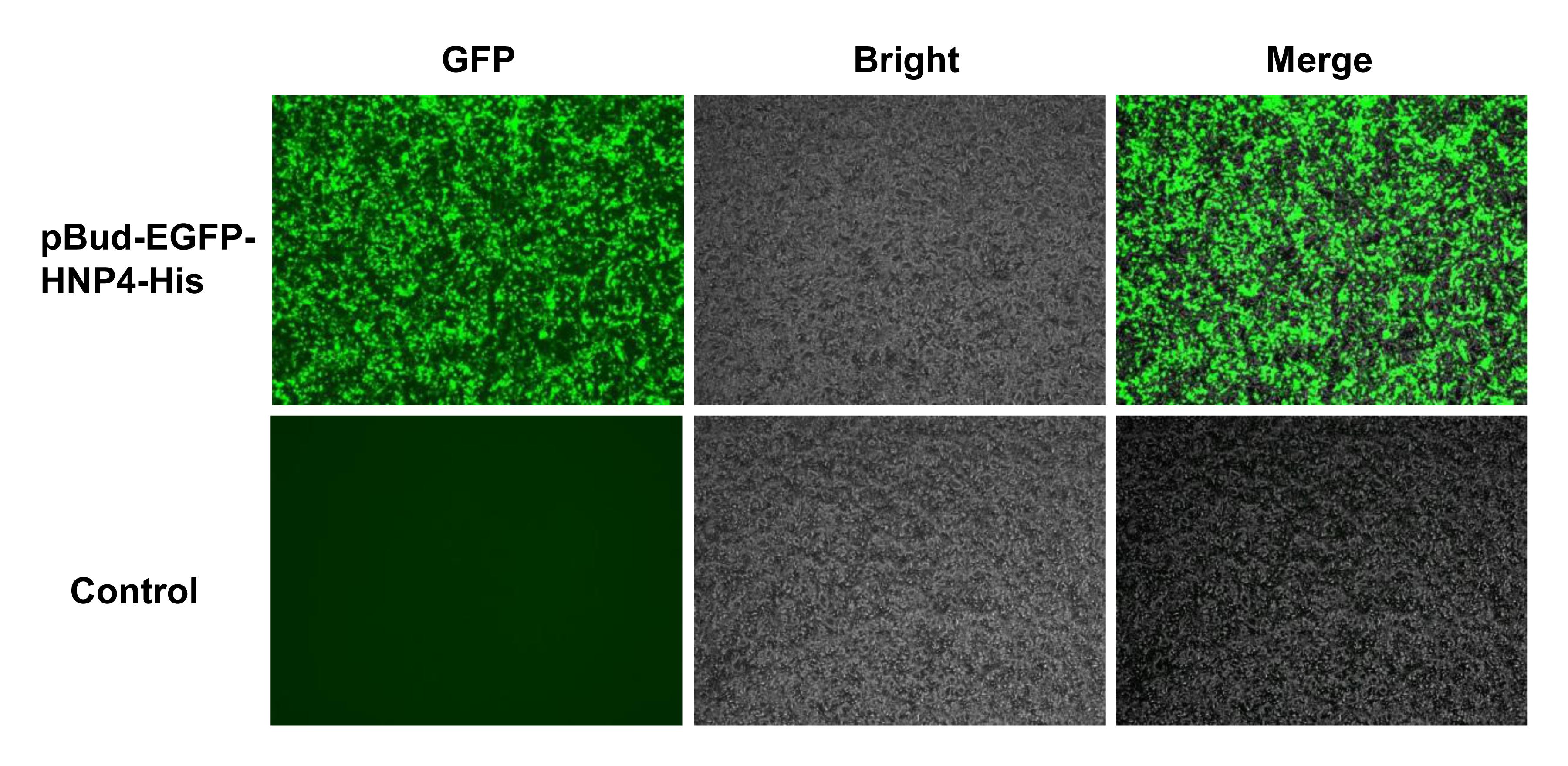

Supplement: S1 Fig — 293T cells were transfected with pBud-EGFP-HNP4-His, and control cells were transfected with an empty vector. GFP was expressed in the transfected cells after incubation for 24 h. (TIF) [file pone.0127922.s001.tif]

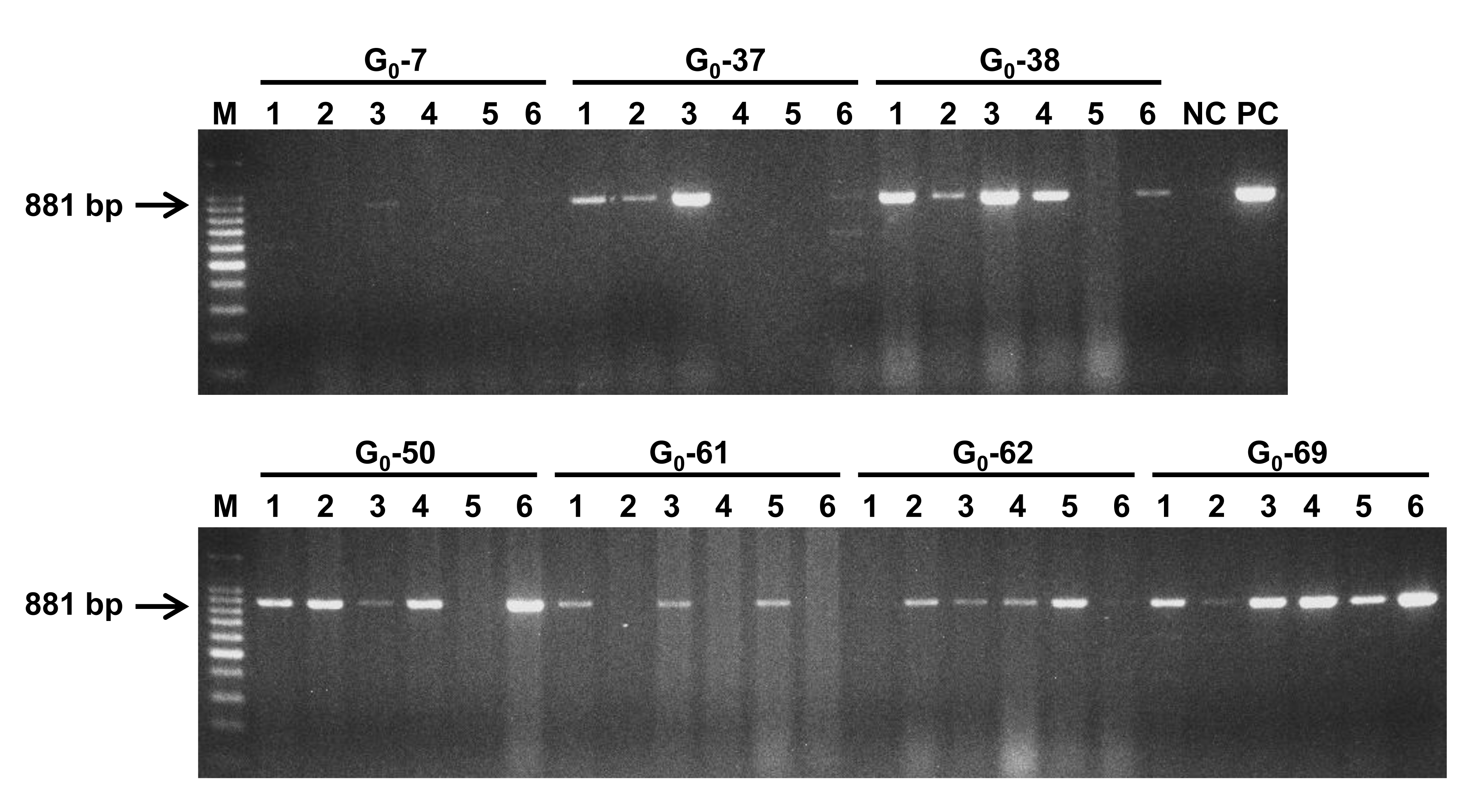

Supplement: S2 Fig — The primers used to screen the transgenic chickens generated an 881-bp PCR product. The figure shows some of the G0 hens detected using this method; the G0 hens shown in the figure are G0-7, G0-37, G0-38, G0-50, G0-61, G0-62, and G0-69. The amount of DNA from G0 chickens used for PCR was 1 μg. M, 100-bp ladder; 1, heart; 2, liver; 3, spleen; 4, lung; 5, kidney; and 6, ovary; NC, negative control (oviduct of the wild-type hen); and PC, positive control (plasmid). (TIF) [file pone.0127922.s002.tif]

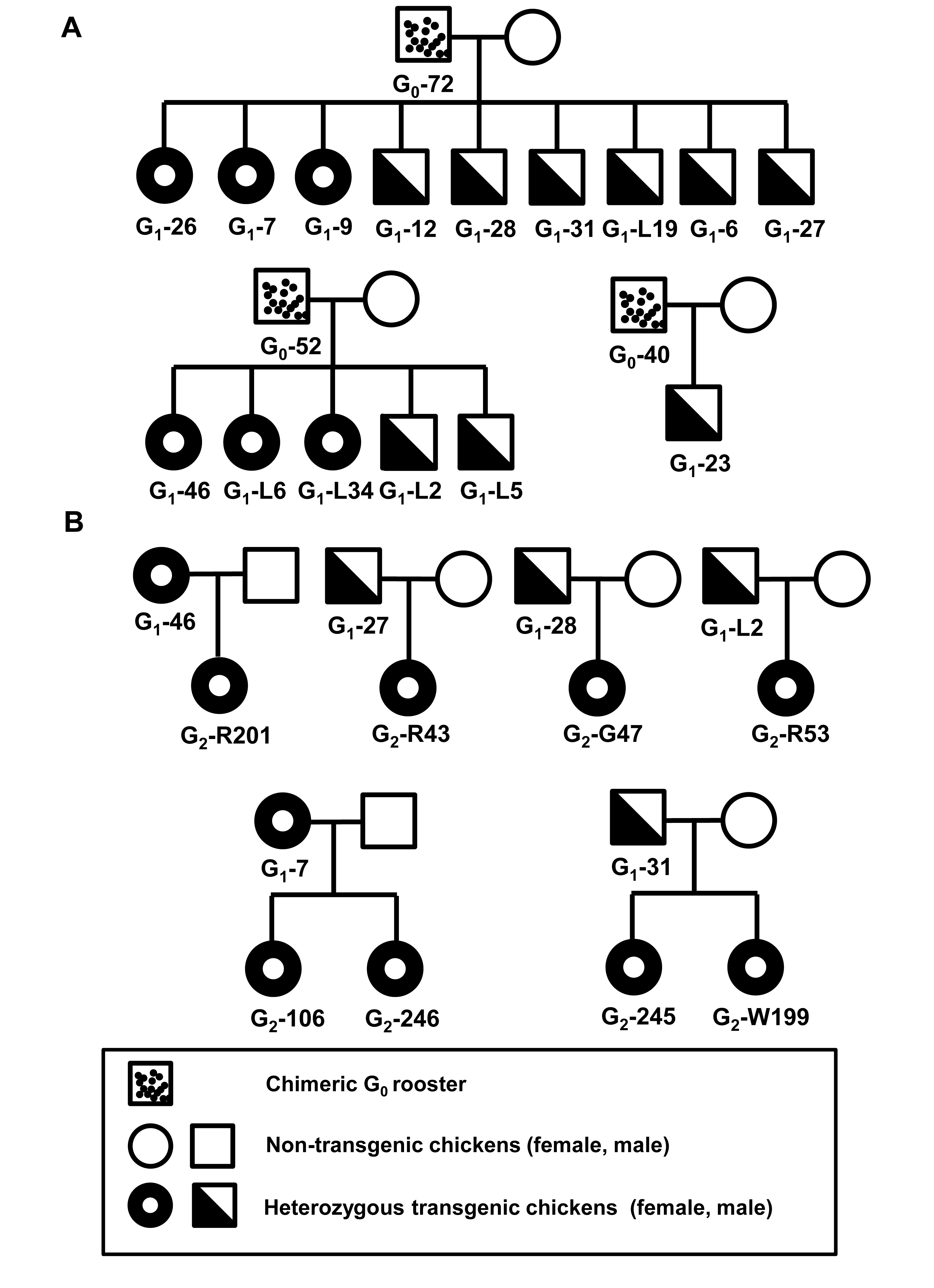

Supplement: S3 Fig — A. Three G0 chimeric chickens (G0-72, G0-52, and G0-40) were crossed with nontransgenic hens to produce the G1 hemizygous chickens. In total, 15 G1 chickens (G1-26, G1-7, G1-9, G1-12, G1-28, G1-31, G1-L19, G1-6, G1-27, G1- 46, G1-L6, G1-L34, G1-L2, G1-L5 and G1-23) were obtained. B. The G2 chickens were obtained using the same methods. (TIF) [file pone.0127922.s003.tif]

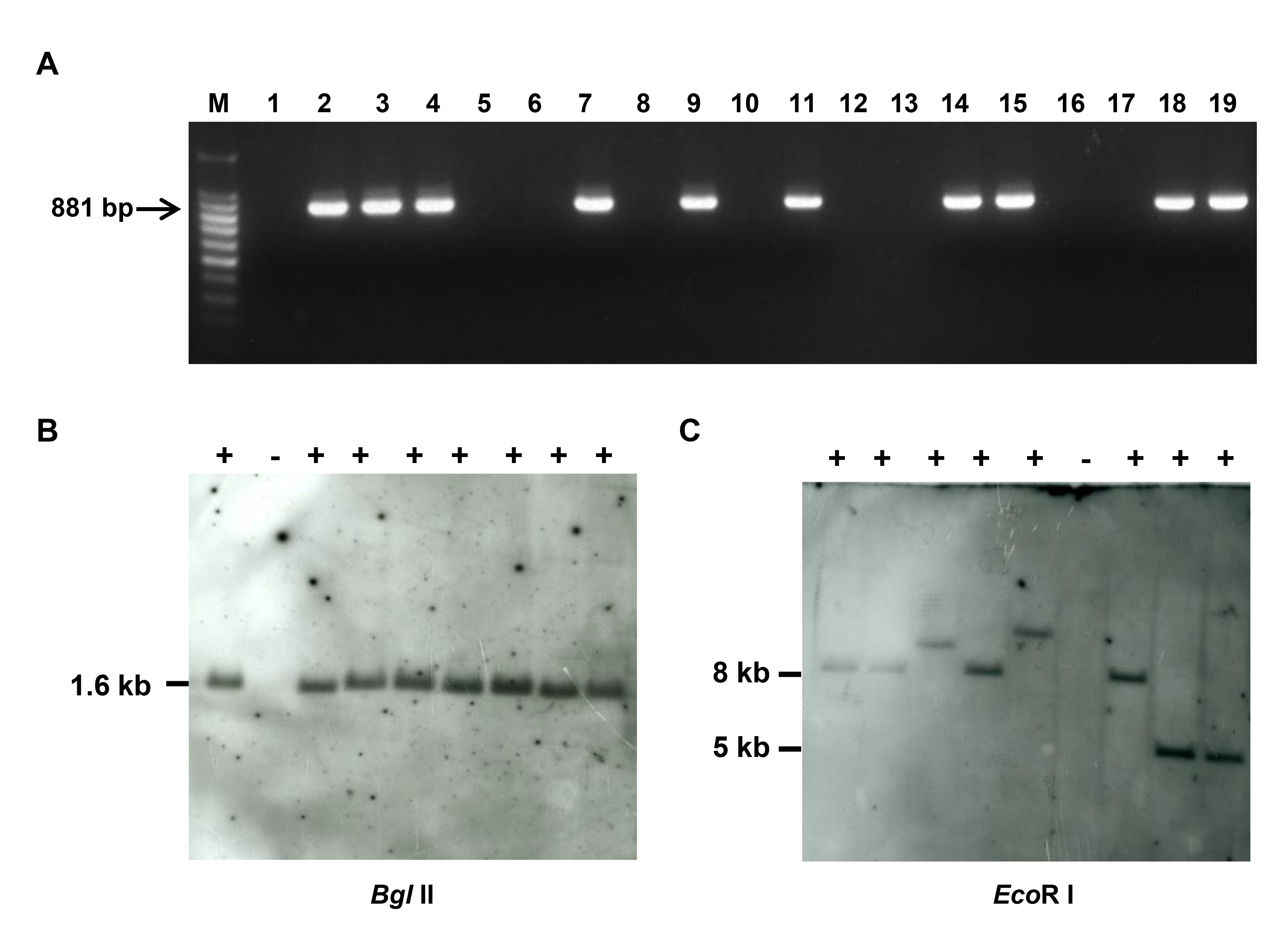

Supplement: S4 Fig — A. PCR analysis of the G2 transgenic chickens. Lane 1, Marker, and Lanes 2–19, DNA samples isolated from the combs of G2 transgenic chickens derived from G1-positive chickens. B and C. Southern blot analysis of the G2 transgenic chickens. “+” represents positive G2 chickens, and “-” represents nontransgenic chickens. The genomic DNA samples were digested with Bgl II (B) and EcoR I (C) to confirm the integration and the copy number of the transgene in the G1 transgenic chickens. (TIF) [file pone.0127922.s004.tif]

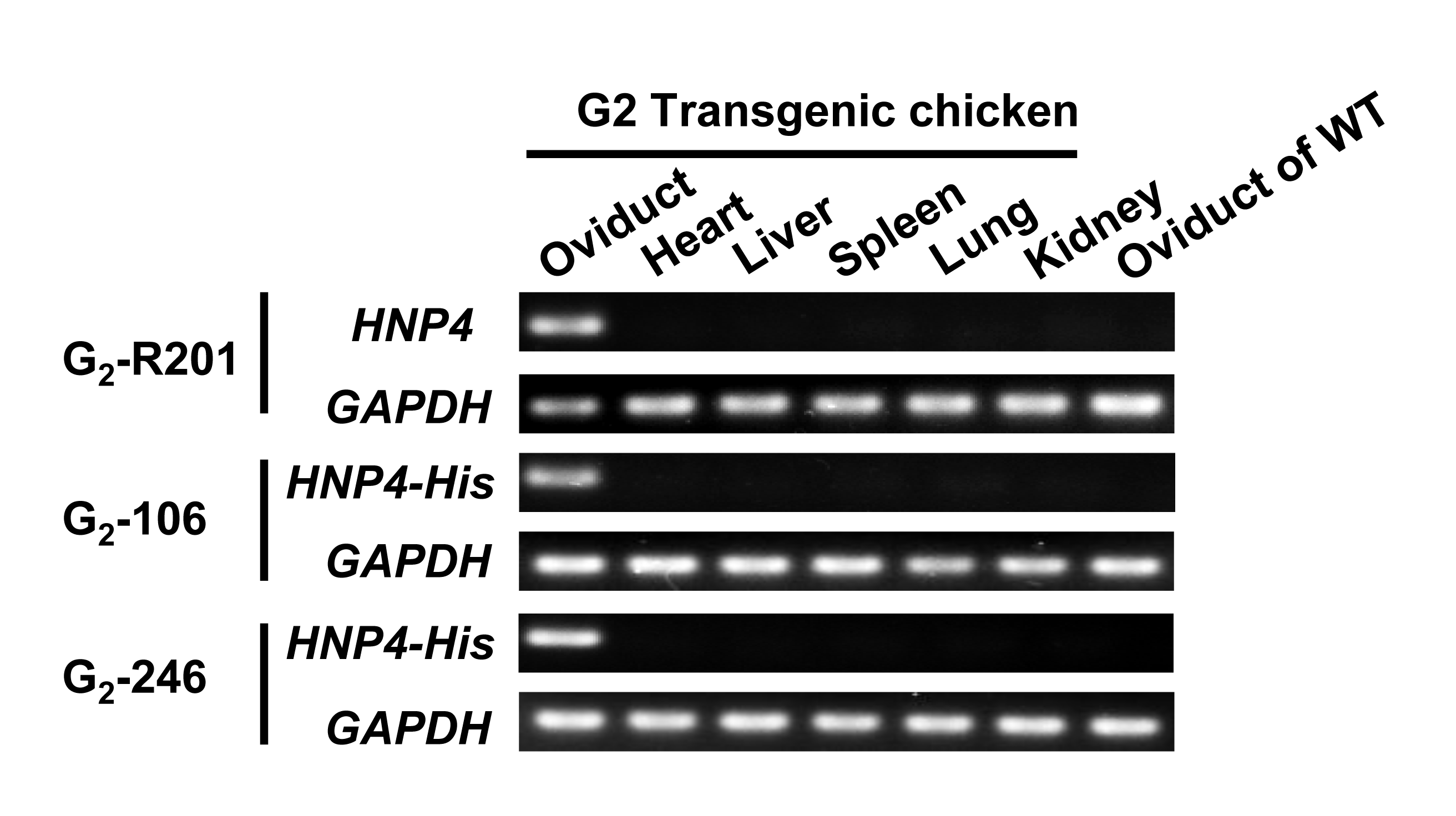

Supplement: S5 Fig — RT-PCR analysis of the expression of the HNP4 and HNP4-His genes in the G2 transgenic hens (G2-R201 with HNP4 and G2-106 and G2-246 with HNP4-His). Lanes 1–6 represent the tissues of the oviduct, heart, liver, spleen, lung and kidney in the transgenic hens (G2), respectively. Lane 7 represents the oviduct tissue in the wild-type hens. (TIF) [file pone.0127922.s005.tif]

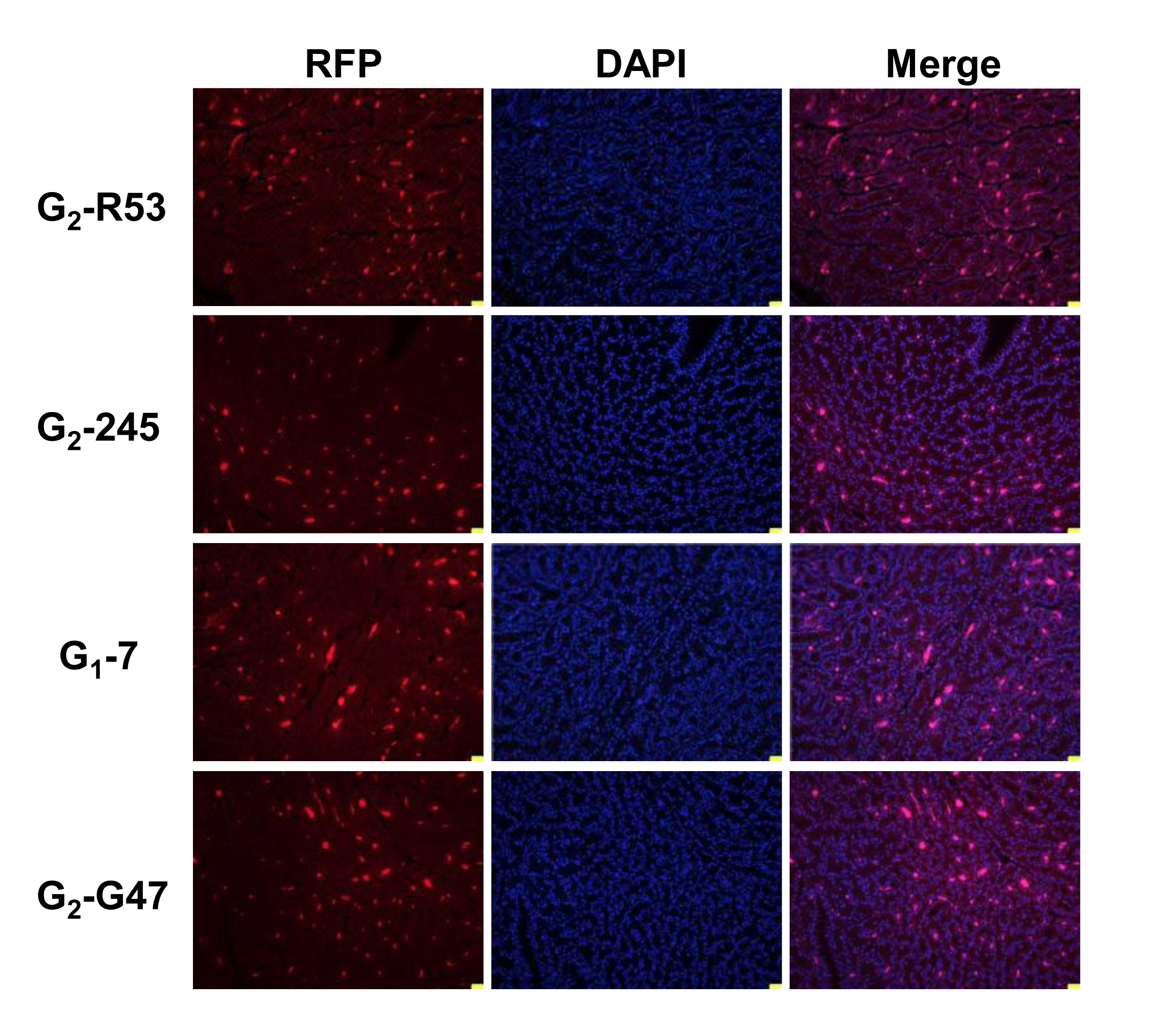

Supplement: S6 Fig — HNP4 protein (red) in the oviduct tissues of hens G2-R53, G2-245, G1-7 and G2-G47 was visualized in the sections of the magnum portion of the oviducts by staining with the HNP4 antibody. (TIF) [file pone.0127922.s006.tif]
