# Supplementary material for: Oviduct-Specific Expression of Human Neutrophil Defensin 4 in Lentivirally Generated Transgenic Chickens
Source: PLoS One. 2015 May 28;10(5):e0127922. doi: 10.1371/journal.pone.0127922 (PMC4447378; doi:10.1371/journal.pone.0127922)
Supplement: S1 Table — (DOCX) [file pone.0127922.s007.docx]

**Table S1. Primers list**

| Primers | Primer sequence |
| --- | --- |
| HNP4-His-sense | 5’GTCTTTGCTAATCTTGGTGCTT3’ |
| HNP4-His-antisense | 5’GAAAGACACGCCGCCAAT3’ |
| Probe-sense | 5’ACTGTGTTTGCTGACGCAAC3’ |
| Probe-antisense | 5’CAACACCACGGAATTGTCAG3’ |
| Primer-F | 5’TTGAGCAAAGCAAACCATACCTGAA3’ |
| Primer-R | 5’GCAAAGACCTCATGGATCCCGTAG3’ |
| RT-HNP4-sense | 5’TCTCGACGGTATCGGTTAACTT3’ |
| RT-HNP4-antisense | 5’GCTAGTCTCGTGATCGGAAAAT3’ |
| RT-GAPDH-F | 5’CGATCTGAACTACATGGTTTACATGTT3’ |
| RT-GAPDH-R | 5’CCCGTTCTCAGCCTTGACA3’ |
