# Supplementary material for: Oviduct-Specific Expression of Human Neutrophil Defensin 4 in Lentivirally Generated Transgenic Chickens
Source: PLoS One. 2015 May 28;10(5):e0127922. doi: 10.1371/journal.pone.0127922 (PMC4447378; doi:10.1371/journal.pone.0127922)
Supplement: S2 Table — (DOCX) [file pone.0127922.s008.docx]

**Table S1. Lentiviral vector injection into chicken embryos**

| Number of injected eggs | Number of hatched chickens | Vctors constructed | Viral titer (IFU/ml) |
| --- | --- | --- | --- |
| 54 | 20 | Ova-HNP4 | 10^8^ |
| 48 | 18 | Ova-HNP4 | 10^8^ |
| 53 | 21 | Ova-HNP4 | 10^9^ |
| 51 | 6 | Ova-HNP4 | 10^10^ |
| 43 | 20 | Ova-HNP4-His | 10^9^ |
| 45 | 3 | Ova-HNP4-His | 10^9^ |
| 50 | 22 | Ova-HNP4-His | 10^9^ |
| 48 | 10 | Ova-HNP4-His | 10^10^ |
| 40 | 12 | Ova-HNP4-His | 10^10^ |
| 45 | 10 | Ova-HNP4-His | 10^10^ |
| 45 | 12 | Ova-HNP4-His | 10^9^ |
| 50 | 14 | Ova-HNP4-His | 10^9^ |
| 47 | 28 | Ova-HNP4-His | 10^9^ |
| 50 | 22 | Ova-HNP4-His | 10^9^ |
